# Monthly Variation, Environmental Drivers, and Ecological Functions of Marine Bacterial Community in a Eutrophic Coastal Area of China

**DOI:** 10.3390/microorganisms13040837

**Published:** 2025-04-07

**Authors:** Zezheng Yan, Yanjian Jin, Tiejun Li, Xiaoling Zhang, Qiao Yang, Chengzhe Ren, Ling Qiao

**Affiliations:** 1 College of Marine Science & Technology, Zhejiang Ocean University, Zhoushan 316004, China; zzyan1198@163.com (Z.Y.); zhangxiaoling@zjou.edu.cn (X.Z.); qiaoyang1979@whu.edu.cn (Q.Y.); 2 Marine Ecological and Environmental Monitoring Center of Zhejiang Province, Zhoushan 316021, China; jinyanjian@126.com; 3 Key Laboratory of Sustainable Utilization of Technology Research for Fishery Resource of Zhejiang Province, Zhejiang Marine Fisheries Research Institute, Zhoushan 316021, China; litiejun1982@126.com

**Keywords:** bacterial communities, monthly variations, environmental factors, surface seawater, ecological functions

## Abstract

This study investigated the monthly variations of bacterial communities in the surface seawater of the Wenzhou coastal area and their influencing factors, while exploring the ecological functions of microbial communities. The results indicated that the surface seawater bacterial communities in this region exhibited high diversity, with significantly higher diversity observed in the winter half-year compared to the summer half-year. The bacterial community structures showed distinct monthly variations, with high similarity between adjacent months, particularly from June to September. The dominant bacterial taxa primarily included Proteobacteria represented by the *SAR86 clade*, *OM43 clade*, and Rhodobacteraceae; Bacteroidota represented by Flavobacteriaceae; and Cyanobacteria mainly composed of *Cyanobium PCC-6307* and *Synechococcus CC9902*. Temperature and nitrate ions were identified as the environmental factors most strongly correlated with monthly bacterial community variations, while dissolved oxygen, nitrite ions, and total organic carbon also showed significant correlations with relative abundances of certain taxa. Predictions of the bacterial community’s ecological functions revealed that chemoheterotrophic functions were most abundant throughout the year, whereas photoautotrophic functions were primarily enriched in summer. Denitrification and other nitrogen cycling-related functions also displayed obvious monthly variations. Collectively, this study provides valuable insights into the temporal changes in coastal microbial communities and their interactions with different environments.

## 1. Introduction

The ocean harbors approximately 10^29^ bacterial and archaeal cells [1]. These microorganisms, characterized by their immense biomass, rapid turnover rates, and complex community structures, serve as critical components in supporting global biogeochemical cycles and maintaining ecosystem stability [2]. On the one hand, marine microorganisms act as key producers and decomposers in ecosystems. For instance, Cyanobacteria convert CO_2_ into oxygen and organic matter through oxygenic photosynthesis. The decomposition and sedimentation of organic carbon contribute to carbon sequestration. Heterotrophic bacteria assimilate labile dissolved organic matter (LDOM) to synthesize biomass, sustaining microbial loops while significantly enhancing marine carbon sequestration through the production of refractory dissolved organic matter (RDOM) [3]. On the other hand, marine microorganisms play pivotal roles in nitrogen cycling. Nitrogen-fixing bacteria such as *Trichodesmium* and *Richelia* convert atmospheric N₂ into bioavailable nitrogen, initiating biological nitrogen cycling [4]. Following organismal death, organic nitrogen is mineralized into ammonium via ammonification, subsequently oxidized to NO_3_^−^ through nitrification, and ultimately reduced back to N_2_ through denitrification and anaerobic ammonium oxidation. Additionally, microorganisms drive the biogeochemical cycles of phosphorus, sulfur, iron, and other elements. For example, Rhodobacteraceae may mediate the conversion of phosphate esters into bioavailable dissolved inorganic phosphorus, suggesting their potential significance in marine phosphorus cycling [5]. Sulfur-metabolizing bacteria such as *Desulfobacter*, *Desulfococcus 1pr3*, and *Desulfobacterium* generate sulfides via the disproportionation of elemental sulfur, sulfite, and thiosulfate [6]. Members of the Zetaproteobacteria oxidize ferrous iron (Fe^2+^) to obtain metabolic energy while promoting iron mineralization [7].

Marine microbial communities exhibit pronounced spatiotemporal variations. On the one hand, their composition shows distinct regional disparities. For example, dominant phyla in the southern Black Sea include Proteobacteria, Cyanobacteria, Bacteroidetes, Planctomycetes, Verrucomicrobiota, and Actinobacteria [8], while surface seawater communities in the Arabian Sea are primarily composed of Firmicutes, Bacteroidetes, *Bacillus*, Rhodobacteraceae, and *Marinobacter* [9]. On the other hand, seasonal shifts in community structure are equally evident. In the northern Yellow Sea marine ranching area (China), Cyanobacteria dominate in summer, whereas Crenarchaeota and Thermoplasmatota exhibit higher relative abundances in winter [10]. The coastal waters of the northern Bohai Sea (China) show elevated abundances of Actinobacteria during dry seasons and Proteobacteria during rainy seasons [11]. In the Yangtze River Estuary, the relative abundance of Actinobacteriota is significantly higher in autumn than in summer, while Cyanobacteria dominate in summer compared to autumn [12].

Environmental parameter changes such as temperature, salinity, dissolved oxygen, light, nutrients, and ocean currents that accompany seasonal shifts are important factors influencing temporal variations in the microbial community structure and diversity. Temperature regulates bacterial growth rates, while salinity shifts alter species composition, thereby influencing community stability and diversity [7,13]. For instance, temperature serves as a key driver of seasonal alpha diversity variations in China’s Shantou coastal area, where summer and winter exhibit the most distinct community compositions. In the Antarctic marginal ice zones, freeze–thaw cycles induce abrupt temperature and salinity changes, driving microbial communities to develop high physiological adaptability through dynamic ribosomal/protein abundance, enzymatic activity, and fatty acid content adjustments, with concomitant shifts in community structure [14]. In the North Atlantic fjords, the seasonal daylight duration shows significant negative correlations with the richness and evenness of euphotic zone microbial communities [15]. Declining seawater DO levels typically promote dominance by anaerobic and facultative anaerobic bacteria, enhancing processes like denitrification and anaerobic ammonium oxidation, which collectively reshape microbial community structures and ecological functions [16].

Nutrient availability further modulates aquatic microbial assemblages. Anthropogenic nitrogen inputs, for example, increase mangrove microbial community richness, diversity, and evenness, while elevating the relative abundance of nitrogen-cycling functional bacteria [17]. However, some studies indicate that elevated nitrate concentrations primarily affect community composition without significantly altering diversity [18]. In summary, marine microbial communities exhibit distinct spatiotemporal variations, with environmental parameters serving as key drivers of these dynamics. Nevertheless, due to the complexity of marine ecosystems and microbial interactions, a consensus remains elusive. Further systematic investigations are urgently required to advance the understanding of marine microbial community dynamics, as well as their environmental drivers and ecological roles.

Among China’s four major coastal seas (the Bohai Sea, Yellow Sea, East China Sea, and South China Sea), the East China Sea is the only typical subtropical sea. The Wenzhou coastal area, located in the southern part of the East China Sea, exhibits distinct seasonal environmental characteristics. This region is abundant in marine organisms including fish, shellfish, and algae, supporting a thriving aquaculture industry. However, intensive fishing, frequent shipping activities, and substantial terrestrial pollutant inputs have resulted in recurrent summer and autumn algal blooms, establishing it as a classic eutrophic marine area. Compared to well-studied regions such as the Bohai Sea, Yellow Sea, Yangtze River Estuary, and South China Sea, research on microbial community structure and ecological functions in the Wenzhou coastal waters remains scarce, particularly regarding the monthly variations, environmental drivers, and associated ecological roles. This study conducted monthly seawater sampling over one year in the Wenzhou coastal waters, analyzed the microbial community structure, investigated their monthly variations and influencing factors, and predicted the ecological functions. The findings aim to provide a reference point for studies on the monthly variations in microbial community structure and ecological functions in eutrophic coastal waters, as well as their environmental drivers.

## 2. Materials and Methods

### 2.1. Study Area and Sampling

**Figure 1:**
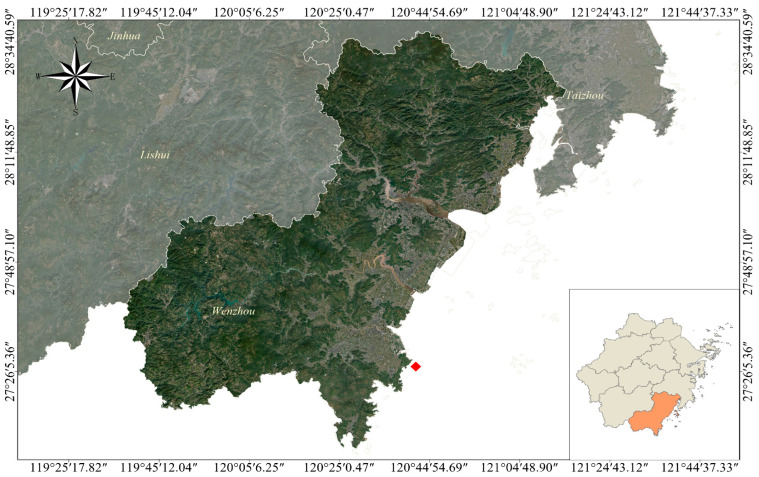
The red dots indicate the sampling locations. Water samples were collected monthly at a fixed sampling point, with consistent sampling conducted at a depth of 1 m below the water surface.

Wenzhou is located in the southeastern part of Zhejiang Province, China, adjacent to the East China Sea. It lies between 27°03′–28°36′ N and 119°37′–121°18′ E. This region falls within the subtropical monsoon climate zone; the water temperature in the coldest month remains above 0 °C. The water sampling site was located in the eutrophic waters off the coast of Wenzhou (Figure 1). The experiment commenced in April 2021 and concluded in March of the following year, during which the surface seawater was collected monthly. To minimize potential data bias in the sequencing results, the samples were collected at consistent sampling locations and processed in strict adherence to the national standard sampling protocols.

The water samples were filtered through a 0.22-micron pore membrane, and the filters were placed in sterile centrifuge tubes, immediately frozen using dry ice, and subsequently stored at −40 °C upon return to the laboratory. Concurrently, field measurements of water temperature, salinity, dissolved oxygen concentration, and pH were conducted using a multiparameter water quality meter. Upon returning to the laboratory, the water samples were analyzed for TOC, NO_3_^−^, NO_2_^−^, NH_4_^+^, DON, PO_4_^3−^, and DTP. TOC was measured using the non-dispersive infrared absorption method; NO_3_^−^, NO_2_^−^, and NH_4_^+^ were measured by continuous flow colorimetry; and DON and DTP were measured through potassium persulfate oxidation.

### 2.2. DNA Extraction, Amplification, and High-Throughput Sequencing

Total community genomic DNA was extracted using the FastDNA™ Spin Kit for Soil (MP Biomedicals, Santa Ana, CA, USA). The quality of the extracted genomic DNA was evaluated by 1% agarose gel electrophoresis, and the DNA concentration and purity were determined using the NanoDrop2000 spectrophotometer (Thermo Scientific, Waltham, MA, USA).

Using the extracted DNA as a template, the V3-V4 hypervariable region of the 16S rRNA gene was amplified by PCR with barcoded primers 338F (5′-ACTCCTACGGGAGGCAGCAG-3′) and 806R (5′-GGACTACHVGGGTWTCTAAT-3′) [19]. Three replicates were performed during PCR amplification. The PCR products were separated on a 2% agarose gel, and the target bands were extracted and purified using the AxyPrep DNA Gel Extraction Kit (AXYGEN, San Francisco, CA, USA). The purified PCR products were quantified using the QuantiFluor^TM^-ST Blue Fluorescence Quantification System (Promega, Madison, WI, USA). Subsequently, purified amplicons were pooled in equimolar.

Library construction was performed using the NEXTFLEX Rapid DNA-Seq Kit according to the following steps: (1) adapter ligation; (2) removal of self-ligated adapter fragments using magnetic bead screening; (3) enrichment of library templates by PCR amplification; and (4) recovery of PCR products using magnetic beads to obtain the final library. Sequencing was carried out on the Illumina PE300 platform (Shanghai Majorbio Bio-pharm Technology Co., Ltd., Shanghai, China). Raw sequencing data were deposited in the NCBI SRA database (Accession Number: PRJNA1221100).

### 2.3. Data Processing and Information Analysis

Quality-filtered sequences were clustered into OTUs at 97% similarity and chimeras were removed using UPARSE v11 [20,21]. Amplicon sequence variants (ASVs) with a total abundance of below 0.005% were filtered out to perform stringent noise filtering. After chloroplast and mitochondrial sequences were excluded, all the samples were normalized to the minimum sequence count, achieving an average Good’s coverage of 97.69%. OTUs were taxonomically annotated using the RDP classifier [22] against the Silva 16S rRNA database (v138) with a 70% confidence threshold, and community composition was analyzed at different taxonomic levels. Alpha diversity indices including Chao, Shannoneven, and Shannon, were calculated at the OTU level. Differences among multiple groups were tested using the Kruskal–Wallis rank-sum test, with multiple testing correction performed using the False Discovery Rate (FDR) method. A post hoc analysis was conducted using the Tukey–Kramer method with a 95% confidence interval.

Hierarchical clustering analysis of all the samples at the OTU level was performed using the Unweighted Pair Group Method with Arithmetic Mean (UPGMA) algorithm. Principal Component Analysis (PCA) was conducted at different taxonomic levels to explore intergroup differences. The Adonis test was used to assess the variation among samples based on the experimental design. The Bray–Curtis distance algorithm was used for similarity measurement.

Redundancy Analysis (RDA) and Canonical Correspondence Analysis (CCA) were employed to analyze the correlations between the samples and the environmental factors. Partial Monte Carlo permutation tests were utilized to evaluate the contribution of each candidate environmental variable in explaining species variables. Spearman’s rank correlation coefficients were calculated to assess the relationships between the environmental factors and the microbial taxa.

The Functional Annotation of Prokaryotic Taxa (FAPROTAX), a manually constructed database based on cultured representatives and literature, was used to map prokaryotic taxa to metabolic or ecologically relevant functions. A functional prediction of the microbial community was performed using the FAPROTAX (version 1.2.1) database, and differences in the predicted functions between the groups were statistically tested. Spearman’s correlation analysis was performed to assess the relationships between species abundance and ecological functions.

## 3. Results

### 3.1. Monthly Variation of Environmental Variables

The physicochemical parameters of the surface seawater measured in this study exhibited obvious monthly variations. The results showed that the seawater temperature (T) ranged from 9.4 to 28.9 °C throughout the year, with an average of 20.42 °C. The lowest T was recorded in February, while the highest was observed in September. Salinity ranged from 23.4 to 31.7, with an average of 27.0. The highest salinity occurred in March, and the lowest was recorded in February. Dissolved oxygen (DO) concentrations ranged from 7.28 to 11.14 mg/L, with an average of 9.08 mg/L. The highest DO concentration was observed in February, while the lowest was recorded in June. The pH values ranged from 7.85 to 8.21, with an average of 8.00. The highest pH was observed in September, and the lowest was recorded in October.

Nutrient concentrations in the water also showed monthly variations. Total organic carbon (TOC) ranged from 1.79 to 5.71 mg/L, with an average of 3.14 mg/L. The highest TOC concentration was observed in February, while the lowest was recorded in September. Nitrate (NO_3_^−^) concentrations ranged from 0.01 to 0.35 mg/L, with an average of 0.18 mg/L. The highest NO_3_^−^ concentration was observed in February, while the lowest was recorded in July. Nitrite (NO_2_^−^) concentrations ranged from 0.002 to 0.249 mg/L, with an average of 0.04 mg/L. The highest NO_2_^−^ concentration was observed in April, while the lowest was recorded in July and August. Ammonium (NH_4_^+^) concentrations ranged from 0.21 to 0.34 mg/L, with an average of 0.27 mg/L. The highest NH4⁺ concentration was observed in January of the following year, while the lowest was recorded in April. Dissolved organic nitrogen (DON) ranged from 1.80 to 7.13 mg/L, with an average of 3.14 mg/L. The highest DON concentration was observed in September, while the lowest was recorded in November. Phosphate (PO_4_^3−^) concentrations ranged from 0.001 to 0.035 mg/L, with the highest concentration observed in August and the lowest in June. Dissolved total phosphorus (DTP) ranged from 0.03 to 0.12 mg/L, with an average of 0.016 mg/L. The highest DTP concentrations were observed in July and October, while the lowest was recorded in May (Table 1). The annual average N/P ratio of the water was 174, indicating a typical phosphorus-limited marine area.

Among the environmental factors, DO showed a significant negative correlation with T (R = −0.860, *p* < 0.01), which is commonly observed in surface seawater due to the decisive influence of temperature on DO solubility. DON exhibited a significant positive correlation with S (R = 0.736, *p* < 0.01), while NO_3_^−^ showed a significant negative correlation with temperature (T) (R = −0.783, *p* < 0.01). These correlations may be attributed to factors such as terrestrial inputs and environment–microorganism interactions.

### 3.2. Bacterial Diversities and Their Temporal Variations

The annual average values of Chao, Shannoneven, and Shannon were 1449, 0.69, and 4.47, respectively, with relative standard deviations of 51.1%, 7.0%, and 14.0%. The Kruskal–Wallis rank-sum test results showed no significant differences in alpha diversities among seasons; however, the Chao and Shannon indices were significantly higher in the winter half-year (October to March of the following year) than in the summer half-year (April to September) (*p* < 0.05). The diversity was highest in November and December and lowest in May, August, and September.

**Figure 2:**
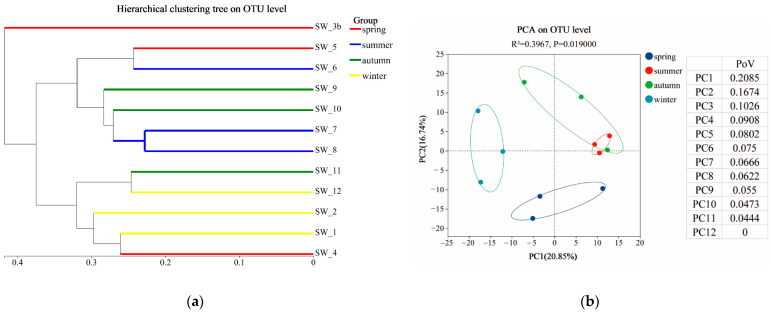
Beta diversity of marine microbial communities: (**a**) cluster analysis at the OTU level; (**b**) PCA analysis at the OTU level. The inter-group R^2^ value was 0.3967 (*p* = 0.019, *p* < 0.05). Principal component 1 (PC1) explained 20.85% of the variance, while principal component 2 (PC2) accounted for 16.74% of the variance (PoV: Proportion of Variance). The samples are designated as SW_4 (corresponding to April), SW_5 (May), and so forth up to SW_12 (December); SW_1, SW_2, and SW_3b represent samples from the following year’s January, February, and March, respectively. The same convention applies to the figure below.

The beta diversity of the microbial communities was explored using hierarchical clustering analysis and Principal Component Analysis (PCA). The clustering analysis results indicated that the communities did not strictly cluster by season, but most adjacent months were closely grouped. The microbial communities were broadly divided into the following two clusters: one from May to October and the other from November to February of the following year and April. The communities in March were distantly grouped from the other months (Figure 2a). Principal Component Analysis (PCA) was conducted on the seawater samples grouped by season, and the Adonis test was used to evaluate the significance of the inter-group differences. The results revealed closer distances between the summer and autumn sample points, indicating more similar microbial community compositions during these two seasons (*p* < 0.05) (Figure 2b).

### 3.3. Microbial Community Structures and Their Monthly Variations

**Figure 3:**
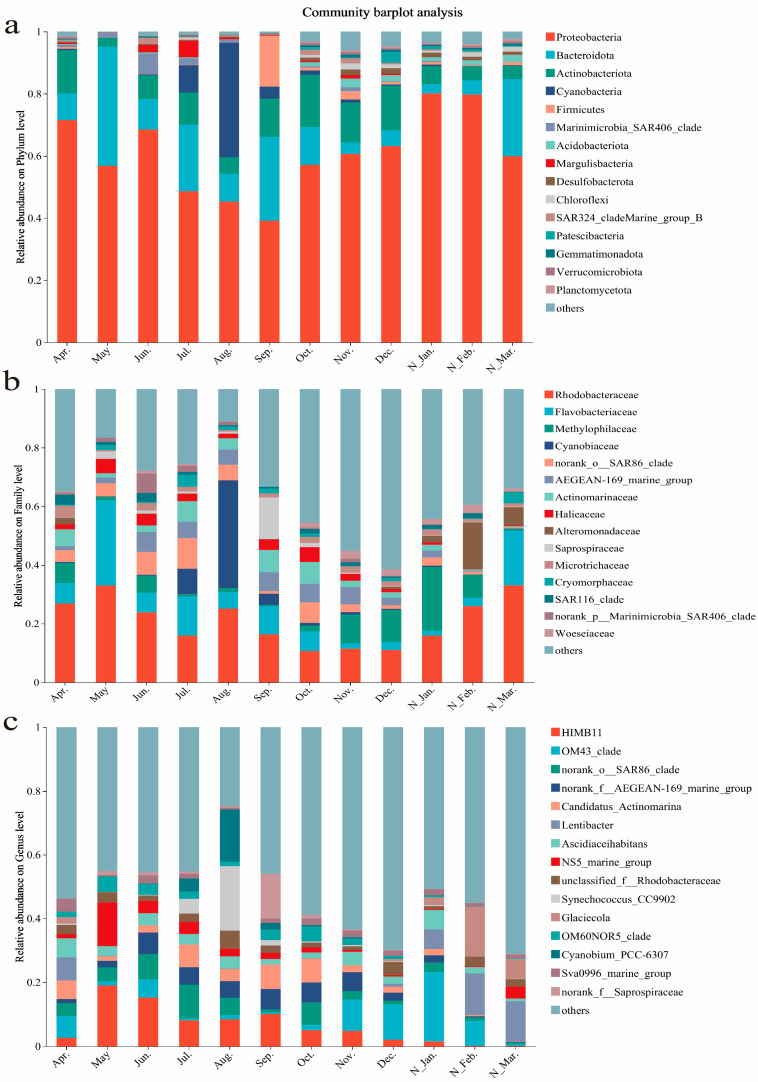
Microbial Community Composition: (**a**) Phylum; (**b**) Family; (**c**) Genus.

The dominant microbial taxa in seawater samples throughout the year were as follows: Proteobacteria (60.89%), Bacteroidota (13.95%), Actinobacteriota (9.25%), Cyanobacteria (4.45%), and Firmicutes (1.98%) were dominant at the phylum level; Rhodobacteraceae (20.80%), Flavobacteriaceae (8.80%), Methylophilaceae (5.80%), Cyanobiaceae (4.38%), and norank_o__SAR86 clade (4.01%) were dominant at the family level; *HIMB11* (6.46%), *OM43 clade* (5.76%), *norank_o__SAR86 clade* (4.01%), *norank_f__AEGEAN-169 marine group* (3.73%), and *Candidatus Actinomarina* (3.50%) were dominant at the genus level (Figure 3).

**Figure 4:**
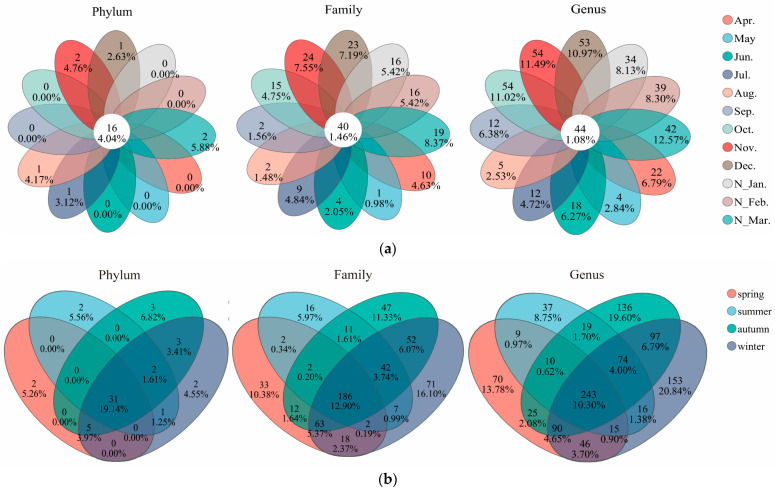
Venn diagrams of microbial community composition at the Phylum, Family, and Genus levels: (**a**) analyze by month; (**b**) analyze by season.

The microbial community composition exhibited pronounced monthly variations. Venn diagram results revealed that the shared phyla, families, and genera across all the months only accounted for 4.04%, 1.46%, and 1.08%, respectively (Figure 4a), while the shared phyla, families, and genera across all the seasons accounted for 19.14%, 12.90%, and 10.30%, respectively (Figure 4b). Thus, the proportion of shared microbial groups decreased from the phylum level to the family level and further to the genus level, with the shared phyla across the months being significantly lower than those across the seasons. At the genus level, the shared taxa across all the months accounted for only 1.08%, while the unique taxa in the individual months ranged from 2.53% to 12.57%, indicating pronounced monthly variations in microbial community structure.

At the phylum level, the bacterial groups shared across all the months with relative abundances >5% included Proteobacteria (63.54%), Bacteroidota (14.56%), and Actinobacteriota (9.65%). At the family level, the taxa shared across all the months with relative abundances >5% were Rhodobacteraceae (28.06%), Flavobacteriaceae (11.88%), Methylophilaceae (7.83%), Cyanobiaceae (5.91%), norank_o__SAR86 clade (5.40%), and AEGEAN-169 marine group (5.03%). At the genus level, the taxa shared across all the months with relative abundances >5% included *HIMB11* (11.28%), *OM43 clade* (10.06%), *norank_o__SAR86 clade* (7.00%), *norank_f__AEGEAN-169 marine group* (6.52%), *Candidatus Actinomarina* (6.11%), and *Ascidiaceihabitans* (5.68%). For seasonal comparisons, at the phylum level, the bacterial groups shared across all the seasons with relative abundances >5% included Proteobacteria (60.99%), Bacteroidota (13.98%), and Actinobacteriota (9.26%). At the family level, the taxa shared across all the seasons with relative abundances >5% were Rhodobacteraceae (21.72%), Flavobacteriaceae (9.19%), and Methylophilaceae (6.06%). At the genus level, the taxa shared across all the seasons with relative abundances >5% included *HIMB11* (7.15%) and *OM43 clade* (6.38%).

The dominant microorganisms in the seawater samples were screened at different taxonomic levels, and the Kruskal–Wallis H test was used for an inter-monthly significance testing of the relative abundance differences. Proteobacteria was the most abundant phylum, accounting for up to 80.11% in the January samples and a minimum of 39.15% in the September samples. Within Proteobacteria, Alphaproteobacteria and Gammaproteobacteria dominated, contributing 36.47% and 29.85% of total bacterial community abundances, respectively, but their relative abundances showed distinct inter-monthly variations. Gammaproteobacteria reached its highest abundance (54.92%) in the January samples and lowest (12.37%) in the August samples. Among Gammaproteobacteria, the SAR86 clade order, *OM43 clade* genus, *OM60NOR5 clade* genus, and *Glaciecola* genus exhibited higher relative abundances. Specifically, SAR86 clade showed significantly higher abundances from December to March and in September (*p* value = 0.004); *OM43 clade* displayed significantly higher abundances from November to February (*p* value = 0.007); *OM60NOR5 clade* had lower abundances in winter (*p* value = 0.033); and *Glaciecola* genus showed significantly higher abundances from November to April but extremely low levels in the other months (*p* value < 0.005).

Alphaproteobacteria accounted for up to 41.13% of the bacterial communities in the March samples and a minimum of 23.86% in the August samples. Rhodobacteraceae was the dominant family within Alphaproteobacteria, with the combined relative abundances of the genera *Ascidiaceihabitans*, *HIMB11*, and *Lentibacter* comprising approximately one-quarter of total community abundances. Specifically, *HIMB11* showed significantly higher relative abundances in the summer half-year compared to the winter half-year (*p* value = 0.01). In contrast, *Lentibacter* exhibited the opposite pattern, with significantly higher abundances from January to April compared to other months (*p* value = 0.006). *Ascidiaceihabitans* maintained stable relative abundances across all months. Members of the SAR11 clade order, also classified under Alphaproteobacteria, accounted for an average of only 2.47% of bacterial community abundances and showed no distinct seasonal patterns.

Flavobacteriaceae was the dominant family within Bacteroidota, contributing approximately 7% to the community’s relative abundance. Both Flavobacteriaceae and Bacteroidota showed lower relative abundances from November to February, with the highest abundance observed in May. Actinobacteriota exhibited higher relative abundances from September to December and in April (>10%), with Acidimicrobiia being the dominant class within this phylum. The genus *Candidatus Actinomarina* accounted for 6.03% of the community’s relative abundance, with a higher abundance observed in April and from July to October (*p* value = 0.004).

Cyanobacteria were primarily composed of two genera, *Cyanobium PCC-6307* and *Synechococcus CC9902*, with their relative abundances peaking from July to September. In August, the relative abundance of Cyanobacteria reached 36.74%, while it remained relatively low in other months (*p* value = 0.012). Firmicutes were mainly represented by the *Planococcus* genus, which accounted for an average of 1.52% of the microbial community’s relative abundance. Its highest abundance occurred in September, while it remained relatively low in other months. The SAR406 clade is ubiquitous across global oceans; however, its relative abundance only accounted for 1.21% in the present study, peaking in June and exhibiting relatively low levels in the other months.

In addition to the above taxa, some species exhibit extremely high abundance in specific months despite their overall low abundance. For example, the *NS5 marine group* accounted for over 13% of the community in May, while *norank_f__Saprospiraceae* exceeded 14% in September.

### 3.4. Influence of Environmental Variables on Microbial Community

**Figure 5:**
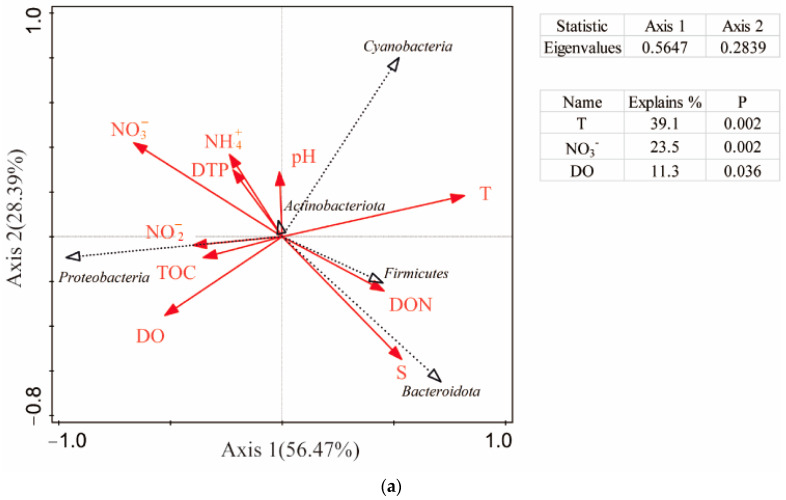

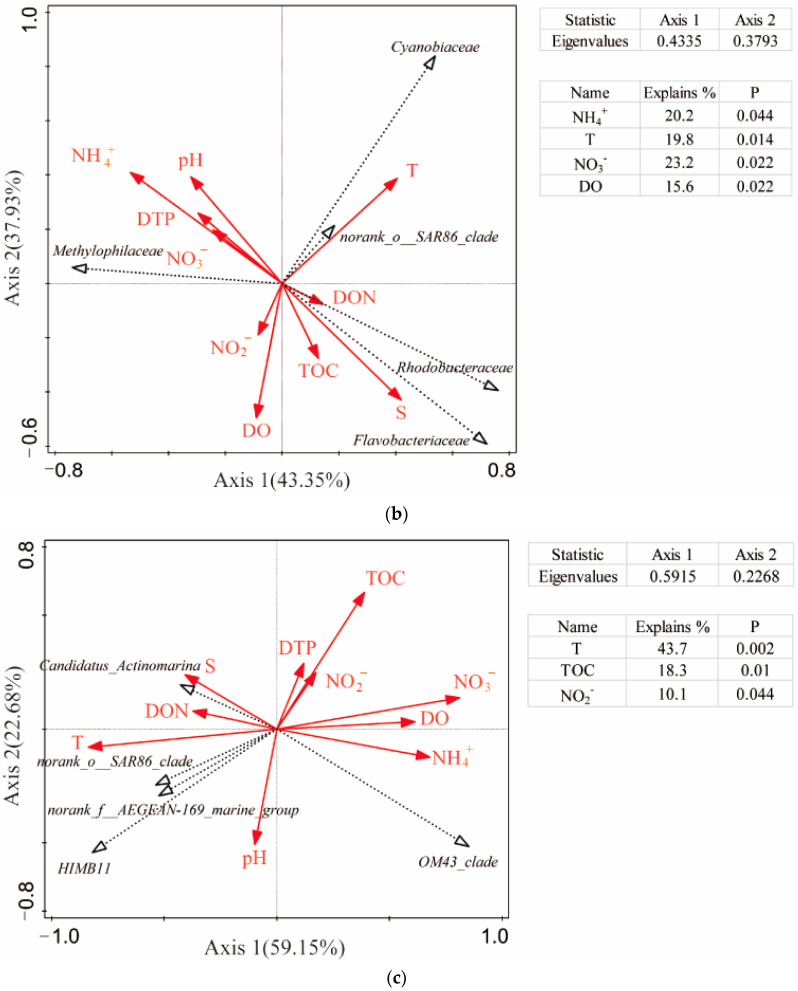
RDA analysis of environmental factors and marine microbial communities: (**a**) Phylum; (**b**) Family; (**c**) Genus.

In this study, Redundancy Analysis (RDA) and Canonical Correspondence Analysis (CCA) were employed to investigate the influence of environmental factors on the structure of seawater microbial communities. The partial Monte Carlo permutation test was used to evaluate the significance of the contribution of individual environmental variables. The results indicated that temperature (T) and nitrate (NO_3_^−^) were likely the most critical factors affecting microbial community structure. In addition, dissolved oxygen (DO), nitrite (NO_2_^−^), and total organic carbon (TOC) also had a certain impact on the community composition. At the phylum level, the seawater samples were significantly influenced by T, NO_3_^−^, and DO (*p* < 0.05), which could explain 38.7%, 23.1%, and 11.4% of the total variations, respectively. At the family level, the seawater samples were significantly influenced by T, NO_3_^−^, DO, and NO_2_^−^ (*p* < 0.05), which could explain 21.7%, 21.7%, 13.0%, and 8.4% of the total variations, respectively. At the genus level, the seawater samples were significantly influenced by T, NO_3_^−^, and TOC (*p* < 0.05), which accounted for 33.1%, 14.3%, and 12.5% of the total variations, respectively (Figure 5).

Spearman’s correlation analysis revealed that the Shannon index was negatively correlated with T and dissolved organic nitrogen (DON) but positively correlated with NO_2_^−^. This suggests that T and different forms of nitrogen (N) significantly influence the microbial community.

**Figure 6:**
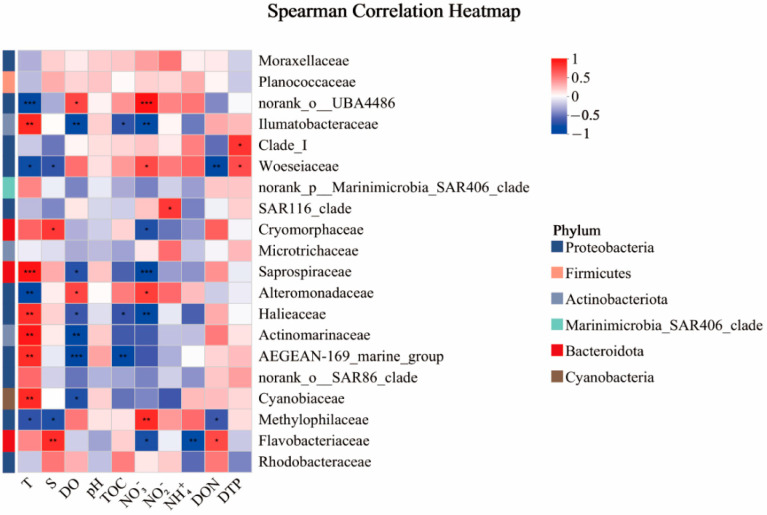
Heatmap of correlation analysis between species and environmental factors at the Family level (* 0.01 < *p* ≤ 0.05, ** 0.001 < *p* ≤ 0.01, *** *p* ≤ 0.001).

Spearman’s correlation analysis was further conducted between the dominant microbial taxa and the environmental factors. For Proteobacteria, the relative abundance of Gammaproteobacteria showed a significant negative correlation with T (R = −0.776, *p* < 0.01). Within Gammaproteobacteria, the *OM43 clade* was negatively correlated with T, salinity (S), and DON but positively correlated with NO_3_^−^. The *OM60NOR5 clade* was positively correlated with T but negatively correlated with NO_3_^−^ and TOC. The *Glaciecola* genus exhibited a highly significant negative correlation with T (R = −0.776, *p* < 0.01) and a significant positive correlation with NO_3_^−^ and TOC. The SAR86 clade showed no significant correlation with the environmental factors. Alphaproteobacteria was significantly negatively correlated with ammonium (NH_4_^+^) (R = −0.734, *p* < 0.01). Within Alphaproteobacteria, Rhodobacteraceae showed no significant correlation with the environmental factors, but *HIMB11* was significantly positively correlated with T (R = 0.818, *p* < 0.01) and negatively correlated with NO_3_^−^ (R = −0.755, *p* < 0.01) and TOC (R = −0.608, *p* < 0.05). In contrast, *Lentibacter* was significantly negatively correlated with T (R = −0.792, *p* < 0.01) but positively correlated with NO_3_^−^ (R = 0.736, *p* < 0.01) and TOC (R = 0.722, *p* < 0.01). Additionally, the SAR11 clade was positively correlated with dissolved total phosphorus (DTP), while *Ascidiaceihabitans* showed no significant correlation with the environmental factors. Bacteroidota was positively correlated with S but negatively correlated with NH_4_^+^ and NO_3_^−^. Its dominant family, Flavobacteriaceae, was positively correlated with S and DON but negatively correlated with NH_4_^+^ and NO_3_^−^. Actinobacteriota was negatively correlated with T and DON, while its dominant class, Acidimicrobiia, and the genus *Candidatus Actinomarina* were significantly positively correlated with T. Cyanobacteria. In addition, its two dominant genera, *Cyanobium PCC-6307* and *Synechococcus CC9902*, were positively correlated with T. Firmicutes. The SAR406 clade showed no significant correlation with the environmental factors. Among taxa with high relative abundances in specific months, the genus *norank_f__Saprospiraceae* was significantly positively correlated with DO, TOC, and NO_3_^−^ but negatively correlated with T. The *NS5 marine group* was significantly positively correlated with T and S but negatively correlated with NO_3_^−^ and NH_4_^+^ (Figure 6, Figures S1 and S2).

### 3.5. Predicted Ecological Functions

**Figure 7:**
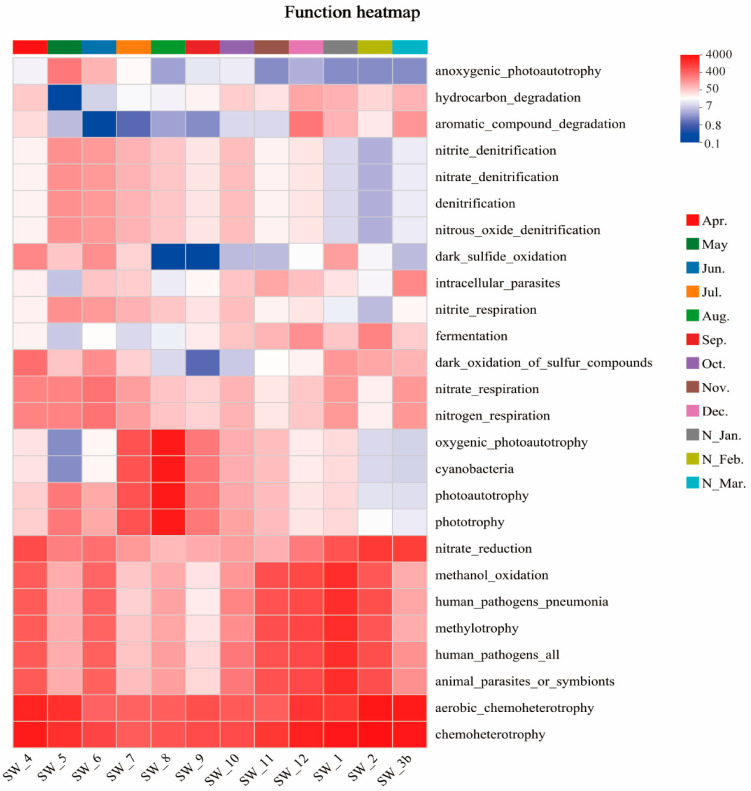
Heatmap of the relative abundance of ecological functions.

The ecological functions of the microbial communities in the Wenzhou coastal waters were predicted using the FARPROTAX (version 1.2.1) database. The results revealed a total of 68 environmental ecological functions associated with the microbial communities in this region. Among these, chemoheterotrophy, oxygenic photoautotrophy, denitrification, sulfur oxidation, hydrocarbon degradation, and parasitism or pathogenicity were identified as the relatively abundant ecological functions in the microbial communities of Wenzhou coastal waters (Figure 7).

**Figure 8:**
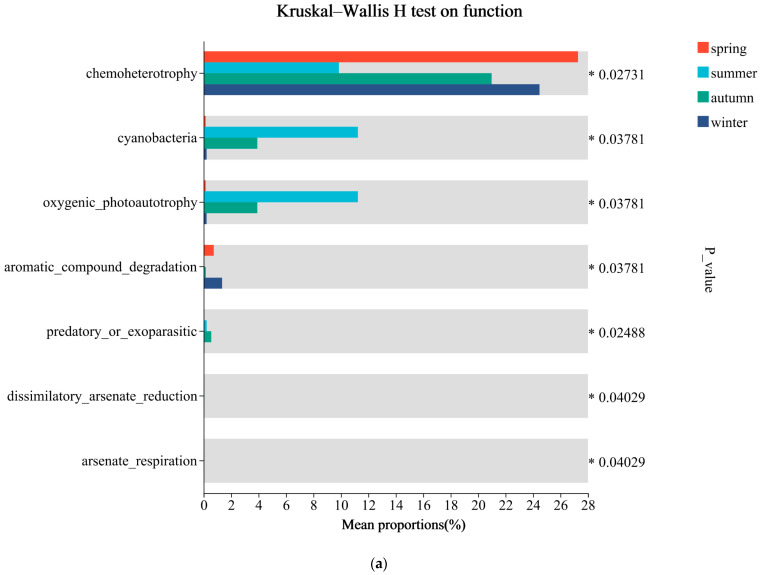

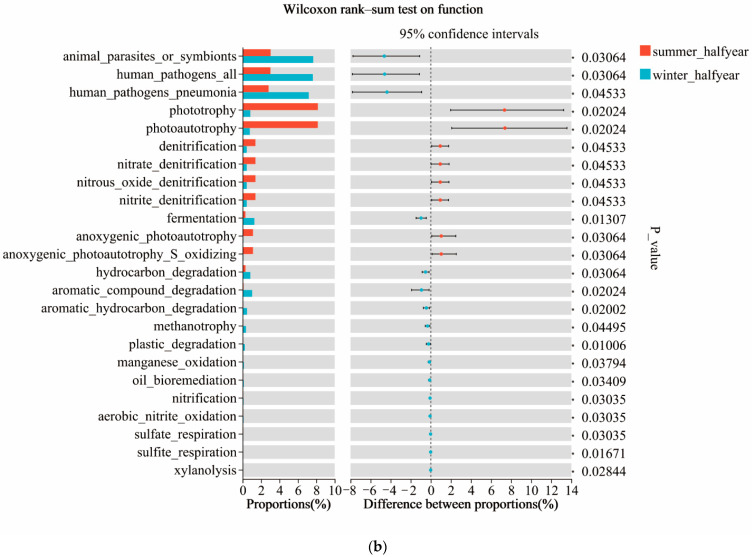
Bar plot of intergroup functional differences (* 0.01 < *p* ≤ 0.05): (**a**) Significant Differences in Ecological Functions Among Different Seasonal Groups; (**b**) Significant Differences in Ecological Functions Between Winter and Summer Half-Year Groups.

The Wilcoxon rank-sum test or Mann–Whitney U test was used to test for differences between the functional groups. Chemoheterotrophy and oxygenic photoautotrophy exhibited significant seasonal variations in relative abundances. Specifically, chemoheterotrophy maintained high relative abundances throughout the year, reaching the highest level in spring, followed by winter, autumn, and summer (*p* < 0.05). Conversely, oxygenic photoautotrophy showed the highest relative abundance in summer, followed by autumn, winter, and spring, peaking in August (*p* < 0.05). This pattern aligned with monthly variations in Cyanobacteria relative abundances (*p* < 0.05). Additionally, the relative abundance of denitrification functions was higher in May and June but lower from January to March (*p* < 0.05). Sulfur oxidation-related functions showed relatively lower abundances in summer and autumn (*p* < 0.05). Relative abundances of certain pathogenic bacteria were higher in the winter half-year compared to the summer half-year, reaching the maximum in January (*p* < 0.05). Similarly, hydrocarbon degradation-related functions also showed higher relative abundances in the winter half-year (*p* < 0.05) (Figure 8).

Among the top 30 most abundant functions, 25 were correlated with water temperature, and 20 were correlated with NO_3_^−^. For instance, functions related to organic matter degradation were generally negatively correlated with water temperature, whereas oxygenic photoautotrophy and denitrification-related functions showed positive correlations with water temperature. Furthermore, human pathogens, methylotrophy, and methanol oxidation functions were negatively correlated with salinity (Table S1).

## 4. Discussion

### 4.1. Characteristics of Microbial Community Diversity and Influencing Factors

High richness, evenness, and diversity indices indicate complex community structures and enhanced ecological stability [23]. In the coastal waters of Wenzhou, the annual mean value of Shannon reached 4.47, comparable to values reported in eutrophic marine ecosystems, including the Yellow Sea coastal surface water (4.97), artificial reef seawater in Laoshan Bay (4.09–5.16), and Hainan coastal aquaculture zones (3.34–4.88) [24,25,26]. Notably, the annual mean Chao index in this study (1449) substantially exceeded those of oligotrophic systems, such as the North Pacific subtropical gyre (97.3), South Pacific subtropical gyre (113.1), and eastern equatorial Pacific (45.5) [27]. These findings suggest that eutrophication levels, particularly nitrogen enrichment in the study area, may drive the observed high alpha diversity of microbial communities.

Temporally, both the Chao and Shannon indices were significantly higher during the winter half-year than in the summer half-year. Raes et al. analyzed the alpha diversity data from eight global observation stations and similarly identified winter peaks in species richness and evenness across tropical, temperate, and polar regions [28]. In the eastern Mediterranean Sea, the Shannon index is lowest during the oligotrophic summer period [29]. In this study, the Shannon index was found to be negatively correlated with water temperature. However, some studies have also demonstrated that the Shannon index of marine microbial communities is lowest in winter and highest in spring, which can be attributed to the influence of water temperature on microbial growth rates [25,30]. For instance, Šolić et al. demonstrated that rising water temperature significantly increased the biomass of heterotrophic microorganisms and picoautotrophic plankton in the eastern Adriatic coastal waters [31]. The primary drivers of microbial alpha diversity remain elusive, as multiple potential parameters may shape community dynamics interactively [32,33]. In this study, the alpha diversity exhibited negative correlations with DON but positive correlations with NO_2_^−^. Notably, despite the phosphorus-limited conditions in the study area, no significant correlations were detected between alpha diversity and PO_4_^3−^, DTP or N/P ratios, suggesting that nitrogen exerts a stronger influence than phosphorus on prokaryotic community diversity. This contrasts with findings from eutrophic waters of the Beibu Gulf, where Shannon indices showed significant positive correlations with dissolved inorganic phosphorus (DIP) and DTP [34]. Furthermore, stochastic processes likely play a substantial role in marine microbial community assembly, warranting further investigation.

### 4.2. Composition and Influencing Factors of Microbial Communities

The dominant bacterial phyla in the study area were Proteobacteria, Bacteroidota, Actinobacteriota, Cyanobacteria and Firmicutes, aligning with the characteristic microbial structure of coastal ecosystems. For instance, a study on surface seawater bacterial communities around Xiamen Island demonstrated the predominance of Proteobacteria, Bacteroidota, Cyanobacteria, and Firmicutes across all sampling stations [35]. Similarly, Jiang et al. reported that microbial communities in the Yangtze River Estuary were primarily dominated by Actinobacteriota, Proteobacteria, Bacteroidota, and Cyanobacteria, reinforcing the prevalence of these phyla in coastal environments [26] (Table 2).

In this study, the sampling and analysis were conducted on a monthly basis, revealing clear distinctions between the monthly and seasonal variation patterns. The proportion of phyla shared among the marine microbial communities across consecutive months was significantly lower than that observed across seasonal intervals. Moreover, the number of taxa unique to each month at the genus level consistently exceeded those shared across months on an annual basis, highlighting pronounced month-to-month structural dynamics in microbial communities. The taxa shared across months at different taxonomic levels exhibited a decreasing trend, indicating significant differences in microbial community structures among samples under environmental changes. Specifically, Proteobacteria was the most abundant taxon at the phylum level. However, within Proteobacteria, the genera *HIMB11*, *Ascidiaceihabitans*, and *OM43 clade* showed distinct environmental adaptabilities at the genus level. *HIMB11* was significantly positively correlated with temperature (T) and negatively correlated with nitrate (NO_3_^−^). In contrast, *OM43 clade* showed opposite correlations with T and NO_3_^−^ compared to *HIMB11*. No significant correlations were observed between *Ascidiaceihabitans* and the environmental factors.

Although the microbial communities did not strictly cluster by season, adjacent months generally showed closer compositional proximity in the cluster analysis. Since environmental parameters can vary significantly between different months within a season, monthly sampling could reflect the variations in microbial community structure and their responses to environmental factors more accurately compared to quarterly sampling. Beta diversity analysis demonstrated greater compositional similarity in warmer periods (June to September or May to October) relative to colder months. Zhang et al. (2022) proposed that under high environmental heterogeneity conditions in low-temperature seasons, heterogeneous selection was the major assembly process, resulting in high β-diversity and more tightly connected co-occurrence networks. However, when environmental heterogeneity decreased in high-temperature seasons, drift took over, leading to a decline in β-diversity and network connectivity [45].

The RDA/CCA results indicated that water temperature and NO_3_^−^ were likely the most critical factors influencing the microbial community structure, with DO, nitrite NO_2_^−^, and TOC also exerting indispensable effects. This result is consistent with the findings reported in the previous studies that focused on coastal microbial communities [34,46,47]. Water temperature is generally considered to be the most critical factor influencing the structure of microbial communities in aquatic ecosystems. For example, 33 OTUs showed significant correlations with water temperature, whereas only 2 OTUs were associated with Chl-a in microbial communities of the Laoshan Bay seawater [25].

Water temperature inherently interacts with multiple ecological characteristics, directly affecting temperature-sensitive microorganisms and indirectly influencing light availability, water stratification, and DO—all of which significantly impact microbial communities [48]. However, in this study, due to the significant correlation between DO and T and the absence of hypoxia in the study area, the actual impact of DO on microbial communities is hypothesized to be limited. Nutrients, particularly nitrogen (N), exhibited pronounced effects on microbial community structure in this region, consistent with findings from the other coastal studies. For instance, nitrate was identified as a key environmental variable affecting bacterial community composition in nori aquaculture waters [49], while turbidity and nitrite were critical factors in the Bohai and North Yellow Sea surface waters [50]. Notably, despite chronic phosphorus limitation (N/P > 34) in the study area, PO_4_^3−^ and DTP showed no significant effects on the microbial community structure or diversity.

Proteobacteria, the most dominant bacterial phylum in global oceans, was prevalent across diverse environments and played vital roles in nutrient cycling. Alphaproteobacteria and Gammaproteobacteria were the most abundant classes in this study, resembling microbial structures in the eutrophic Beibu Gulf waters [34]. However, the low abundance of Deltaproteobacteria may reflect their adaptation to deeper aquatic environments [51]. The relative abundances of Alphaproteobacteria and Gammaproteobacteria exhibited distinct monthly variations with differential responses to environmental factors. The SAR86 clade, *OM43 clade*, *OM60NOR5 clade*, and *Glaciecola* are groups with relatively high abundance within Gammaproteobacteria. SAR86 is an abundant and ubiquitous heterotroph in the surface ocean that plays a central role in marine ecosystem functioning (Global ecotypes in the ubiquitous marine clade SAR86). In phosphorus-limited eastern Mediterranean waters, its abundance can account for over half of the Gammaproteobacteria population [29]. In this study, its abundance was higher in summer–autumn but showed no significant environmental correlations. Both the *OM43 clade* and *Glaciecola* displayed negative correlations with T, peaking in colder months. The *OM43 clade*, a mesophilic and psychrophilic marine group, is generally confined to coastal zones [52,53]. *Glaciecola* genomes contain cold adaptation genes (e.g., EPS synthesis genes), facilitating survival in low-temperature environments [54]. Conversely, the *OM60NOR5 clade* showed minimal abundance and positive T correlation. Overall, Gammaproteobacteria abundance negatively correlated with water temperature, peaking in November–February and declining in July–September. Furthermore, the relative abundances of the *OM43 clade*, *Glaciecola*, and *OM60NOR5 clade* also showed significant correlations with NO_3_^−^, however these correlations were conversely related to their associations with temperature. This dual pattern suggests the following two plausible interpretations: On the one hand, it may indicate direct ecological linkages between NO_3_^−^ and these genera; on the other hand, the observed relationships could alternatively stem from the inherent negative correlation between T and NO_3_^−^ in this system. P. Kalaitzidou studied bacterial communities in water samples from Thermaikos Bay, Greece’s largest mussel farming area, and found *Halomonas* sp., *Sulfitobacter* sp., and *Planococcus* sp. to be the dominant bacterial taxa [55]. In contrast to this study’s results, *Halomonas* sp. and *Sulfitobacter* sp. showed extremely low relative abundances (<0.5%) in our samples, whereas *Planococcus* displayed a high abundance (>9%) in the September samples. *Planococcus* has been closely linked to hydrocarbon degradation capacity and has been isolated from oil-contaminated regions in Iran [56] and petroleum-polluted soils on the Qinghai–Tibet Plateau [57]. These findings suggest that the abnormal presence of *Planococcus* in Wenzhou’s coastal area during this period may indicate oil pollution, potentially associated with intensive shipping activities in the region. However, this hypothesis warrants further investigation.

Compared to Gammaproteobacteria, Alphaproteobacteria exhibited smaller monthly variations. Rhodobacteraceae, a dominant family within Alphaproteobacteria, participates in the assimilation of phytoplankton metabolites and plays significant roles in nutrient cycling and high-molecular-weight (HMW) compound transformation [58]. The combined relative abundances of three genera—*Ascidiaceihabitans*, *HIMB11*, and *Lentibacter*—collectively accounted for approximately one-quarter of the total community abundance. Among these, *HIMB11* showed the highest annual mean abundance, with its relative abundance positively correlated with T, predominantly occurring in samples collected from May to November. During this period, the seawater temperature near Wenzhou averaged above 20 °C, suggesting that elevated temperatures favor *HIMB11* proliferation. Furthermore, the relative abundance of *HIMB11* demonstrated negative correlations with NO_3_^−^ and TOC. Studies have shown that the *HIMB11* strain typically functions as an opportunistic taxon in environmental settings, capable of persisting under oligotrophic conditions until the emergence of favorable conditions for rapid growth (e.g., phytoplankton blooms) [59]. As harmful algal blooms (HABs) frequently occur in coastal waters near Wenzhou from April to October, the high abundance of *HIMB11* observed between May and November might be closely linked to these bloom events. *Lentibacter*, a genus associated with coastal and estuarine waters, has been repeatedly isolated from algal blooms across diverse geographical regions [60,61]. In contrast to *HIMB11*, *Lentibacter* exhibits higher relative abundances from January to April, with lower levels in the other months, showing significant negative correlations with T but positive correlations with NO_3_^−^ and TOC. Although the SAR11 clade is a member of the Alphaproteobacteria, it accounts for only 2.47% of the bacterial community’s relative abundance on average. However, some studies have identified this clade as the most abundant bacterial group in surface ocean waters, comprising 20–24% of communities in South China Sea [62,63] samples, with its dominance more pronounced in oligotrophic regions minimally influenced by human activities [29]. Overall, Rhodobacteraceae reaches its lowest abundance from October to December but shows no significant correlations with environmental factors, while Alphaproteobacteria demonstrates only a significant negative correlation with NH_4_^+^.

Bacteroidetes are recognized as effective utilizers of high-molecular-weight (HMW) organic matter (primarily polysaccharides and proteins) in marine environments, capable of efficiently consuming dissolved organic matter (DOM) and degrading polysaccharides/proteins [64,65]. Flavobacteriaceae, their dominant family, exhibited lower relative abundances from November to February and peaked in May. Hou et al. (2024) observed elevated Bacteroidota abundances during the spring–autumn seasons in Daihai Lake’s littoral microbial communities, showing similarities with the patterns observed in this study [66]. The negative correlations between Flavobacteriaceae and NH_4_^+^, NO_3_^−^ align with their known competitive advantage under mesotrophic and oligotrophic conditions [34]. Similarly, Eilers et al. (2001) demonstrated Bacteroidetes’ survival capabilities during algal blooms in North Sea surface waters, consistent with our finding of their positive correlation with dissolved organic nitrogen (DON) [67]. Actinobacteriota, another dominant phylum in marine microbial communities, showed higher relative abundances (>10%) from September to December and in April, with negative correlations to water temperature and DON. In contrast, Fang et al. (2022) reported that Actinobacteria’s summer dominance was strongly influenced by salinity, diverging significantly from our results [25]. These disparities highlight the critical role of spatial heterogeneity in shaping microbial temporal dynamics and environmental drivers. Due to the complexity of marine ecosystem, ecological patterns identified in one region may not be suitable for other regions.

However, seasonal variations in the abundance of certain microbial taxa still have regional universality, such as Cyanobacteria. As pivotal primary producers in marine ecosystems, Cyanobacteria dominate prokaryotic communities in open oceans, often exceeding 50% relative abundance, yet show reduced proportions in eutrophic coastal waters. In this study, the Cyanobacteria community primarily comprised the following two genera: *Cyanobium PCC-6307* and *Synechococcus CC9902*. Although *Prochlorococcus* is widely recognized as a dominant Cyanobacterial group, its negligible abundance here may reflect its superior adaptation to oligotrophic conditions in open oceans, whereas coastal systems favor *Synechococcus* dominance [68,69,70]. The Cyanobacteria in this study demonstrated positive correlations with water temperature, peaking in relative abundance (36.74%) during July–September and emerging as the dominant phylum in August. This is consistent with the global phenomenon of blue-green algae reaching their peak in summer and decreasing to their lowest point in winter, which is mainly driven by temperature [25,48]. In contrast, *Synechococcus* maintains year-round dominance in the Pearl River Delta [71], likely attributable to stable light/temperature regimes and abundant nutrient. Notably, Cyanobacteria showed no significant correlations with N, P, or TOC, possibly because the nutrients are non-limiting in eutrophic zones and no Cyanobacterial blooms occurred during sampling.

The SAR406 clade, though ubiquitous in global oceans, exhibited low relative abundance here. Studies indicate its prevalence in mesopelagic waters, with abundances fivefold higher than surface layers [72]. While SAR406 dynamics may associate with DO [73], this study found no significant links to temperature, salinity, or nutrients.

### 4.3. Ecological Functions of Microbial Communities and Their Monthly Variations

This study predicted the functional profiles based on the data of the microbial community structure. Previous research has demonstrated that compositional shifts in microbial communities can alter their functional processes, indicating that microbial assemblages are not always functionally redundant [74]. The relatively abundant environmental functions in this study included chemoheterotrophy, photoautotrophy, denitrification, sulfur oxidation, hydrocarbon degradation, and parasitism/pathogen. Correlation analysis between microbial relative abundances and environmental ecological functions revealed significant relationships. Chemoheterotrophy and photoautotrophy were significantly correlated with Proteobacteria and Cyanobacteria. Specifically, Proteobacteria showed a significant positive correlation with chemoheterotrophy (R = 0.811, *p* < 0.01) and a significant negative correlation with photoautotrophy (R = −0.762, *p* < 0.01). Conversely, Cyanobacteria exhibited an opposite pattern, where there were a significant negative correlation with chemoheterotrophy (R = −0.727, *p* < 0.01) and significant positive correlation with photoautotrophy (R = 0.671, *p* < 0.05). Denitrification and sulfur oxidation functions were significantly positively correlated with Bacteroidota (R = 0.618, *p* < 0.05 and R = 0.598, *p* < 0.05, respectively). Hydrocarbon degradation functions showed a significant negative correlation with *HIMB11* (R = −0.832, *p* < 0.01). Parasitic/pathogenic functions were significantly positively correlated with Proteobacteria (R = 0.867, *p* < 0.01) but negatively correlated with Bacteroidota (R = −0.916, *p* < 0.01).

These patterns likely reflect intense anthropogenic pressures in nearshore ecosystems. Elevated proportional abundances of nitrifying bacteria and sewage-/faecal-related taxa have been documented in human-impacted marine regions. The chemoheterotrophic function was associated with ubiquitous heterotrophic bacteria, maintained a consistently high relative abundances throughout the year. Photoautotrophic function peaked in August, aligning with monthly variation patterns of Cyanobacteria dominance. Nitrogen cycling functions were primarily contributed by Gammaproteobacteria and Nitrospirota.

Environmental changes can modulate ecological functional expression by restructuring microbial assemblages. For instance, organic matter degradation-related functions predominantly exhibited negative correlations with water temperature, whereas photoautotrophic processes positively correlated with water temperature. This suggests that monthly variations of water temperature regulate the balance between autotrophic and heterotrophic microorganisms, thereby influencing the production and degradation of aquatic organic matter. Notably, while elevated water temperature may enhance metabolic rates, our functional predictions based solely on bacterial relative abundances did not incorporate such kinetic effects. However, other studies have demonstrated that seasonal shifts (summer vs. winter) in coastal waters can alter predicted microbial metabolisms, with carbohydrate metabolism and membrane transport peaking during winter [75]. In the subtropical regions, the nutrient levels in water bodies are higher during the cold winter and spring seasons, which may facilitate the proliferation of microorganisms.

The relative abundance of aerobic chemoheterotrophy exhibited a significant positive correlation with total organic carbon (TOC), reflecting the microbial community’s immediate response to aquatic TOC levels. Microbial community dynamics may also exert feedback effects on environmental conditions. For instance, denitrification-related functional potentials reached their minimum in February and peaked in May, while nitrate (NO_3_^−^) concentrations peaked in February and declined to their lowest in July. The denitrification potentials showed positive correlations with water temperature but negative correlations with NO_3_^−^. Contrary to expectations that elevated NO_3_^−^ should enhance denitrification, our results demonstrated an inverse pattern. Similarly, the studies in Chesapeake Bay revealed that both denitrifiers and anammox bacteria were positively influenced by temperature and negatively by NO_x_^−^ [76]. Elevated temperatures likely promote denitrifier proliferation, thereby intensifying denitrification efficiency and accelerating nitrate depletion—a mechanism potentially explaining the observed negative correlations between NO_3_^−^ and both denitrification potential and water temperature. This pattern partially reflects the denitrifier’s role in shaping aquatic nutrient profiles. Additionally, the negative correlation between dissolved inorganic nitrogen (DIN) and water temperature may be mediated by photoautotrophic activity. Cyanobacteria proliferate extensively during warm summer months, consuming substantial DIN and reducing its ambient concentrations [76]. This study observed significant negative correlations between NO_3_^−^ and the abundance of photoautotrophy.

The relative abundance of methylotrophy, methane oxidation, and human pathogen-related functions exhibited congruent monthly variation patterns, suggesting their common origins. Their abundances showed negative correlations with salinity, indicating potential contributions from terrestrial inputs. Methylotrophs, a group of bacteria utilizing C1 compounds (e.g., methanol, methane) as sole carbon/energy sources, include environmentally critical taxa like aerobic methanol oxidizers that efficiently degrade methanol under oxic conditions—key agents in bioremediation. Pathogenic microorganisms in marine environments are strongly influenced by anthropogenic activities through multiple pathways: sewage discharge, surface runoff, riverine inputs, groundwater infiltration, and wastewater sludge disposal [77]. Notably, human pathogen-related functions showed a higher abundance primarily during the colder winter months. Billaud’s study indicated that rising seawater temperatures are a major driver of pathogen spread in marine environments, as elevated temperatures favor the growth and reproduction of pathogens (e.g., *Vibrio* spp.) and expand their distribution ranges [78]. Our findings clearly contradict this conclusion. Based on correlations between species abundances and environmental ecological functions, pathogenic taxa were hypothesized to originate from Proteobacteria—a phylum that forms the core of bacterial diversity in marine environments and includes numerous opportunistic and obligate pathogens [79]. Their high winter abundances might be attributed to the Zhe–Min Coastal Current, which flows southward during winter and carries nutrient-rich discharges from the Yangtze River, potentially promoting pathogen proliferation [80]. However, since human pathogen-related functions also peaked in April and June, we cannot exclude the possibility that anthropogenic activities in coastal areas (e.g., ballast water discharge from shipping vessels or spills of transported materials) contributed to these fluctuations.

Furthermore, interconnections exist among the various ecological functions within the microbial communities. For instance, sulfur oxidation showed positive correlations with nitrate reduction. Studies indicate that certain sulfur-oxidizing bacteria can reduce nitrate while oxidizing reduced sulfur compounds. Therefore, monthly environmental variations driving microbial community restructuring may directly influence a suite of interconnected ecological functions, which in turn exert feedback effects on environmental conditions. It should be noted that the factors governing microbial ecological functionalities are highly complex. Our methodology, solely based on community composition for functional prediction, does not account for environmental modulation of metabolic rates or other physiological parameters. Significant differences in community composition and ecological functions of marine microbial communities across months directly influence key ecological processes such as organic matter decomposition and nutrient cycling (e.g., carbon, nitrogen, phosphorus) [81]. For example, the rapid growth of Cyanobacteria and other microorganisms in summer accelerates the air–sea exchange of greenhouse gases like CO_2_ and CH_4_, thereby promoting carbon sequestration [82]. The enhanced activity of denitrifying bacteria (e.g., *OM43 clade*, *Glaciecola*) under low winter temperatures may reduce nitrate accumulation in water through denitrification, alleviating eutrophication [83]. Inter-monthly environmental fluctuations caused by climate change could disrupt microbial community stability (e.g., temperature increases elevate Cyanobacteria and Bacteroidota abundances while reducing Proteobacteria), with shifts in dominant taxa potentially weakening the climate resilience of marine communities [84]. Irregular variations in human pathogen-related pathogenic bacteria resulting from these changes may exacerbate infection risks for fisheries and humans, posing substantial potential threats to ecological stability and human activities [85].

## 5. Conclusions

This study employed high-throughput sequencing to investigate the microbial community characteristics in surface waters of Wenzhou’s coastal zone, revealing pronounced monthly variations in community structure. Dominant taxa included Proteobacteria, Bacteroidota, Cyanobacteria, *HIMB11*, *OM43 clade*, and SAR86 clade. The microbial assemblage exhibited strong correlations with seawater temperature and nitrate concentrations. Functional profiling indicated relatively high metabolic potentials for chemoheterotrophy, photoautotrophy, and denitrification, with these processes being modulated by environmental drivers. Our findings elucidate the monthly dynamics, environmental determinants, and ecological functions of coastal microbial communities near Wenzhou, providing foundational insights for understanding cross-monthly microbial succession and biogeochemical roles in nearshore ecosystems. Future studies will consider expanding the sampling areas and increasing the sample points to investigate the impact of the Zhe–Min Coastal Current on regional microbial communities. Additionally, metagenomic techniques will be employed to explicitly analyze ecological functional genes corresponding to different bacterial taxa, aiming to deepen our understanding of the contributions of diverse bacteria to key biogeochemical processes.

## Figures and Tables

**Table 1:** Environmental variables in the surface water of Wenzhou nearshore area.

Month	T (°C)	S	DO (mg/L)	pH	TOC (mg/L)	NO_3_^−^ (mg/L)	NO_2_^−^ (mg/L)	NH_4_^+^ (mg/L)	DON (mg/L)	DTP (mg/L)	PO_4_^3−^ (mg/L)	N\P
April	17.3	27.8	9.54	7.93	3.69	0.27	0.249	0.21	4.61	0.05	0.033	49
May	22.8	28.6	10.57	7.94	2.89	0.04	0.010	0.22	2.87	0.03	0.01	60
June	23.5	27.0	7.28	7.96	2.00	0.12	0.030	0.22	3.43	0.09	0.001	826
July	28.6	31.3	7.75	8.01	2.94	0.01	0.002	0.26	3.49	0.12	0.01	60
August	28.7	24.3	7.69	8.02	2.74	0.26	0.002	0.28	2.67	0.08	0.035	34
September	28.9	31.7	7.63	8.21	1.79	0.03	0.003	0.27	7.13	0.04	0.014	47
October	24.6	23.5	8.00	7.85	2.39	0.05	0.065	0.25	2.07	0.12	0.005	163
November	20.3	24.3	8.36	8.05	2.56	0.21	0.016	0.30	1.80	0.10	0.027	43
December	15.4	25.4	9.82	8.06	3.78	0.27	0.013	0.33	2.47	0.08	0.028	49
N_January *	12.0	25.3	10.87	7.93	2.28	0.30	0.007	0.34	2.38	0.07	0.025	57
N_February *	9.50	23.4	11.14	8.06	5.71	0.35	0.070	0.28	2.22	0.10	0.005	310
N_March *	13.4	32.0	10.34	7.99	4.96	0.25	0.020	0.26	2.55	0.09	0.003	391
Mean	20.4	27.0	9.08	8.00	3.14	0.18	0.04	0.27	3.14	0.08	0.016	174.18
SD	6.55	3.08	1.38	0.39	1.14	0.12	0.067	0.04	1.41	0.03	0.012	226.29
RSD (%)	311.62	877.56	659.79	1999.8	275.74	153.2	60.92	676.78	223.06	309.79	136.96	76.97

* The month with “N_” in the sampling time was the month of the following year.

**Table 2:** Dominant microbial communities in different regions.

Geographic Location	Dominant Taxa	Influencing Factors	References
Global Distribution	Proteobacteria, SAR11 (Alphaproteobacteria), SAR86 (Gammaproteobacteria); Cyanobacteria, Deferribacteres, Thaumarchaeota;	Light, Temperature, Dissolved Oxygen, Nutrients and Carbon sources	[36]
Arctic Region	Proteobacteria, Bacteriodetes; class level, Alphaproteobacteria, Flavobacteria, Gammaproteobacteria;	Spatial and Seasonal Variability, Nutrients	[37]
Shenzhen Area	Proteobacteria, Actinobacteria, Bacteroidetes; genus level, *Acinetobacter*, *NS5 marine group* (Flavobacteriaceae), *Candidatus Actinomarina*, *HIMB11* (Rhodobacteraceae), *Candidatus Nitrosopumilus*, *Candidatus Aquiluna*, *Aeromonas*, *Cyanobium PCC-6307*, *Arcobacter*, *Synechococcus CC9902* and *Ottowia*, which accounted for 6.2%, 5.9%, 5.8%, 5.8%, 5.0%, 3.7%, 3.1%, 2.1%, 2.0%, 1.7% and1.5%;	Salinity and TN	[38]
Northern Java, Indonesia	Gammaproteobacteria, Alphaproteobacteria and Bacteroidia (38%, 18% and 16%);	Salinity and Temperature	[39]
Dongzhai Bay, Hainan (Aquaculture Zone)	Proteobacteria (59.19–75.19%), Cyanobacteria (6.54–22.84%), Bacteroidetes (8.45–14.51%), Actinobacteria (1.36–11.68%), Marinimicrobia SAR406 clade (0–1.72%), Verrucomicrobia (0.23–1.2%), Epsilonbacteraeota (0.14–1.03%), Euryarchaeota (0–0.72%), Chloroflexi (0–0.64%) and Planctomycetes (0.11–0.56%);	Salinity, Temperature, pH, NH_4_^+^-N, COD, and TN	[40]
Southeastern New South Wales Coast, Australia	Proteobacteria (47.33% ± 2.14), Bacteroidota (21.97% ± 1.35), Cyanobacteria (16.76% ± 2.20), Actinobacteria (11.80% ± 0.84), Verrucomicrobiota (0.56% ± 0.08), Planctomycetota (0.37% ± 0.05), Chloroflexota (0.15% ± 0.04) and SAR324 clade (0.15% ± 0.04);	Seasonal Variability, pH, Salinity, Average depth, Flushing time, and Percentage of the catchment i.e., cleared	[41]
Antarctic Region	Bay: Proteobacteria, Cyanobacteria and Bacteroidota (42.07%, 24.67% and 33.03%); Lake: Bacteroidota, Actinobacteria and Proteobacteria (34.17%, 24.53% and 21.57%);	pH, Salinity, DO, and PO_4_^3−^-P	[42]
Bohai Sea, China	Proteobacteria (45.93–72.9%), Bacteroidetes (15.03–34.65%), Actinobacteria (2.25–13.68%), Cyanobacteria (0.79–10.20%), Planctomycetes (1.46–2.18%), Firmicutes (0.77–3.75%), Acidobacteria (0.78–2.15%), Chloroflexi (0. 28–3.47%);	Nutrients and Dissolved Oxygen	[43]
Yellow Sea	In Sediment: Proteobacteria (47%), Acidobacteria, Actinobacteria, Bacteroidetes, Planctomycetes and Verrucomicrobia; Bacteroides, Myxococcus, Flavobacterium, Planctomycetes and Verrucomicrobium;	TP, Particle Size, TN, and TOC	[44]
Southwestern Coast of India	In the laboratory: Diatoms, Small (<5 µm diameter) flagellated cells and the microzooplankton *Protoperidinium* spp.	Salinity and Temperature	[13]
Wenzhou	Proteobacteria, Bacteroidota, Cyanobacteria, *HIMB11*, *OM43 clade*, and *SAR86 clade*;	Temperature, NO_3_^−^, DO, NO_2_^−^ and TOC	This study

## Data Availability

The raw sequencing reads are available from the NCBI through BioProject accession number PRJNA1221100.

## Supplementary Materials

The following supporting information can be downloaded at: https://www.mdpi.com/article/10.3390/microorganisms13040837/s1, Table S1: Correlation Analysis between Environmental Factors and Ecological Functions; Figure S1: Heatmap of correlation analysis between species and environmental factors at the Phylum level; Figure S2: Heatmap of correlation analysis between species and environmental factors at the Genus level.
