# Supplementary material for: Monthly Variation, Environmental Drivers, and Ecological Functions of Marine Bacterial Community in a Eutrophic Coastal Area of China
Source: Microorganisms. 2025 Apr 7;13(4):837. doi: 10.3390/microorganisms13040837 (PMC12029234; doi:10.3390/microorganisms13040837)
Supplement: Supplementary file 1 [file microorganisms-13-00837-s001.zip › microorganisms-3530759-supplementary.pdf]

Table S1. Correlation Analysis between Environmental Factors and Ecological Functions.

Ecological function	T	S	DO	pH	TOC	NO_3^-	NO_2^-	NH_4^+	DON	DTP	DIN	PO_4^{3-}	N/P
	**Correlation**	**Correlation**	**Correlation**	**Correlation**	**Correlation**	**Correlation**	**Correlation**	**Correlation**	**Correlation**	**Correlation**	**Correlation**	**Correlation**	**Correlation**
dark_sulfite_oxidation	-0.638*	-0.032	0.547	-0.254	0.575	0.755**	0.392	-0.081	0.081	-0.279	0.783**	0.101	0.208
dark_sulfur_oxidation	-0.638*	-0.032	0.547	-0.254	0.575	0.755**	0.392	-0.081	0.081	-0.279	0.783**	0.101	0.208
anoxygenic_photoautotrophy_S_oxidizing	0.534	0.358	-0.434	-0.13	-0.238	-0.634*	-0.039	-0.715**	0.619*	-0.157	-0.569	-0.163	0.203
anoxygenic_photoautotrophy	0.534	0.358	-0.434	-0.13	-0.238	-0.634*	-0.039	-0.715**	0.619*	-0.157	-0.569	-0.163	0.203
hydrocarbon_degradation	-0.636*	-0.109	0.49	-0.217	0.343	0.615*	0.312	0.399	-0.378	0.056	0.643*	0.189	-0.021
aromatic_compound_degradation	-0.788**	-0.128	0.739**	-0.26	0.557	0.715**	0.361	0.35	-0.459	-0.126	0.658*	0.223	-0.039
nitrate_denitrification	0.712**	0.234	-0.579*	-0.062	-0.361	-0.740**	-0.309	-0.474	0.389	-0.021	-0.765**	-0.151	0.07
nitrite_denitrification	0.712**	0.234	-0.579*	-0.062	-0.361	-0.740**	-0.309	-0.474	0.389	-0.021	-0.765**	-0.151	0.07
denitrification	0.712**	0.234	-0.579*	-0.062	-0.361	-0.740**	-0.309	-0.474	0.389	-0.021	-0.765**	-0.151	0.07
nitrous_oxide_denitrification	0.712**	0.234	-0.579*	-0.062	-0.361	-0.740**	-0.309	-0.474	0.389	-0.021	-0.765**	-0.151	0.07
nitrite_respiration	0.734**	0.266	-0.594*	-0.074	-0.378	-0.755**	-0.301	-0.517	0.448	-0.056	-0.762**	-0.154	0.077
fermentation	-0.587*	-0.55	0.413	0.242	0.238	0.601*	0.459	0.517	-0.713**	0.336	0.531	-0.014	0
dark_oxidation_of_sulfur_compounds	-0.629*	0.095	0.399	-0.378	0.301	0.455	0.48	-0.329	0.147	-0.091	0.49	-0.204	0.503
nitrate_respiration	0.007	0.473	-0.098	-0.725**	-0.112	-0.182	0.161	-0.678*	0.462	-0.28	-0.112	-0.246	0.441
nitrogen_respiration	0.007	0.473	-0.098	-0.725**	-0.112	-0.182	0.161	-0.678*	0.462	-0.28	-0.112	-0.246	0.441
cyanobacteria	0.671*	-0.154	-0.608*	0.249	-0.441	-0.294	-0.536	0.301	0.119	0.203	-0.14	0.47	-0.566
oxygenic_photoautotrophy	0.671*	-0.154	-0.608*	0.249	-0.441	-0.294	-0.536	0.301	0.119	0.203	-0.14	0.47	-0.566
photoautotrophy	0.923**	0.098	-0.685*	0.193	-0.531	-0.699*	-0.620*	-0.161	0.434	-0.084	-0.636*	0.225	-0.378

phototrophy	0.923**	0.098	-0.685*	0.193	-0.531	-0.699*	-0.620*	-0.161	0.434	-0.084	-0.636*	0.225	-0.378
nitrate_reduction	-0.804**	0.109	0.629*	-0.312	0.545	0.545	0.595*	-0.217	-0.007	0.007	0.531	-0.404	0.657*
methanol_oxidation	-0.699*	-0.620*	0.455	0.007	0.133	0.804**	0.389	0.483	-0.622*	0.105	0.727**	0.274	-0.098
human_pathogens_pneumonia	-0.734**	-0.637*	0.497	0.021	0.182	0.846**	0.417	0.469	-0.608*	0.112	0.762**	0.235	-0.042
methylotrophy	-0.699*	-0.620*	0.455	0.007	0.133	0.804**	0.389	0.483	-0.622*	0.105	0.727**	0.274	-0.098
human_pathogens_all	-0.790**	-0.578*	0.538	-0.028	0.217	0.839**	0.462	0.441	-0.615*	0.126	0.755**	0.165	0.028
animal_parasites_or_symbionts	-0.790**	-0.578*	0.538	-0.028	0.217	0.839**	0.462	0.441	-0.615*	0.126	0.755**	0.165	0.028
aerobic_chemoheterotrophy	-0.671*	0.06	0.790**	-0.13	0.664*	0.538	0.427	-0.035	-0.091	-0.343	0.49	-0.007	0.154
chemoheterotrophy	-0.958**	-0.161	0.853**	-0.203	0.483	0.769**	0.564	0.14	-0.371	-0.175	0.671*	-0.112	0.294

* 0.01 < P ≤ 0.05, ** 0.001 < P ≤ 0.01, *** P ≤ 0.001.

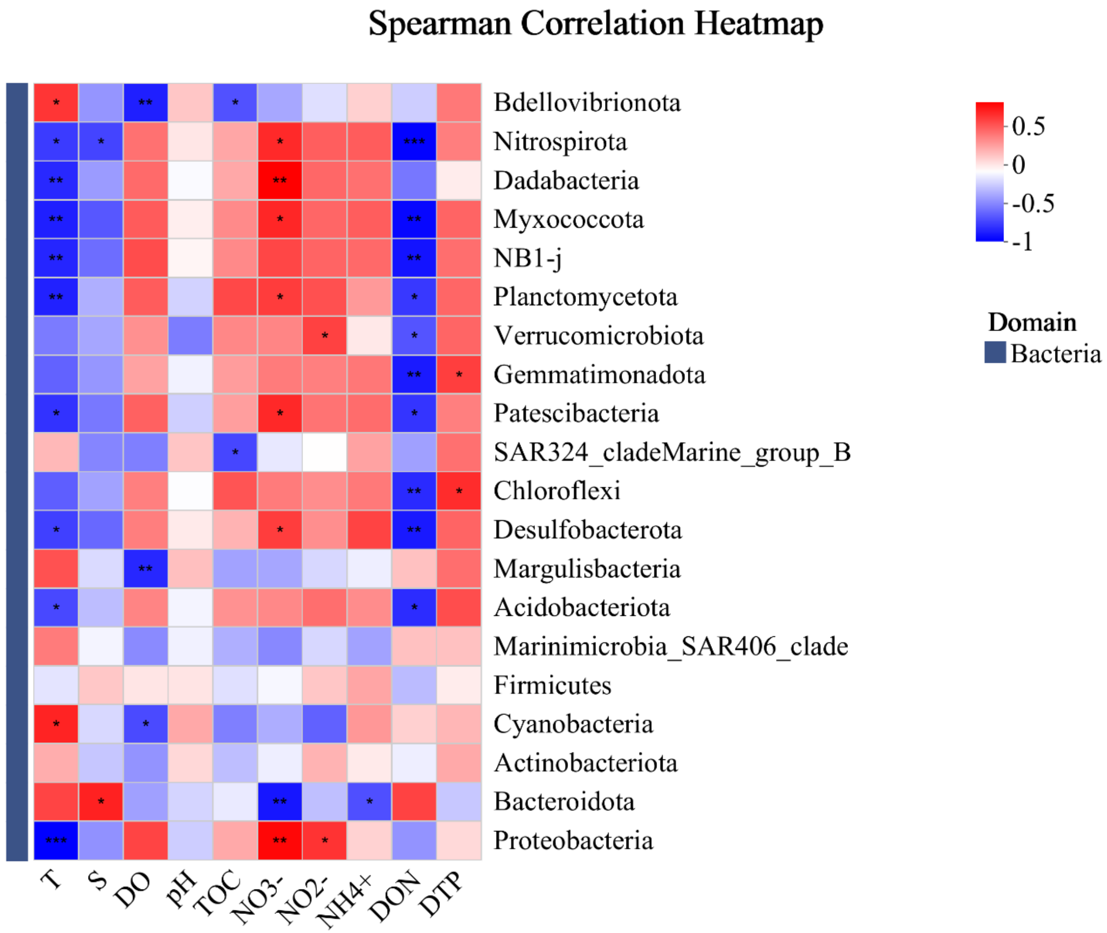

Figure S1. Heatmap of correlation analysis between species and environmental factors at the Phylum level.

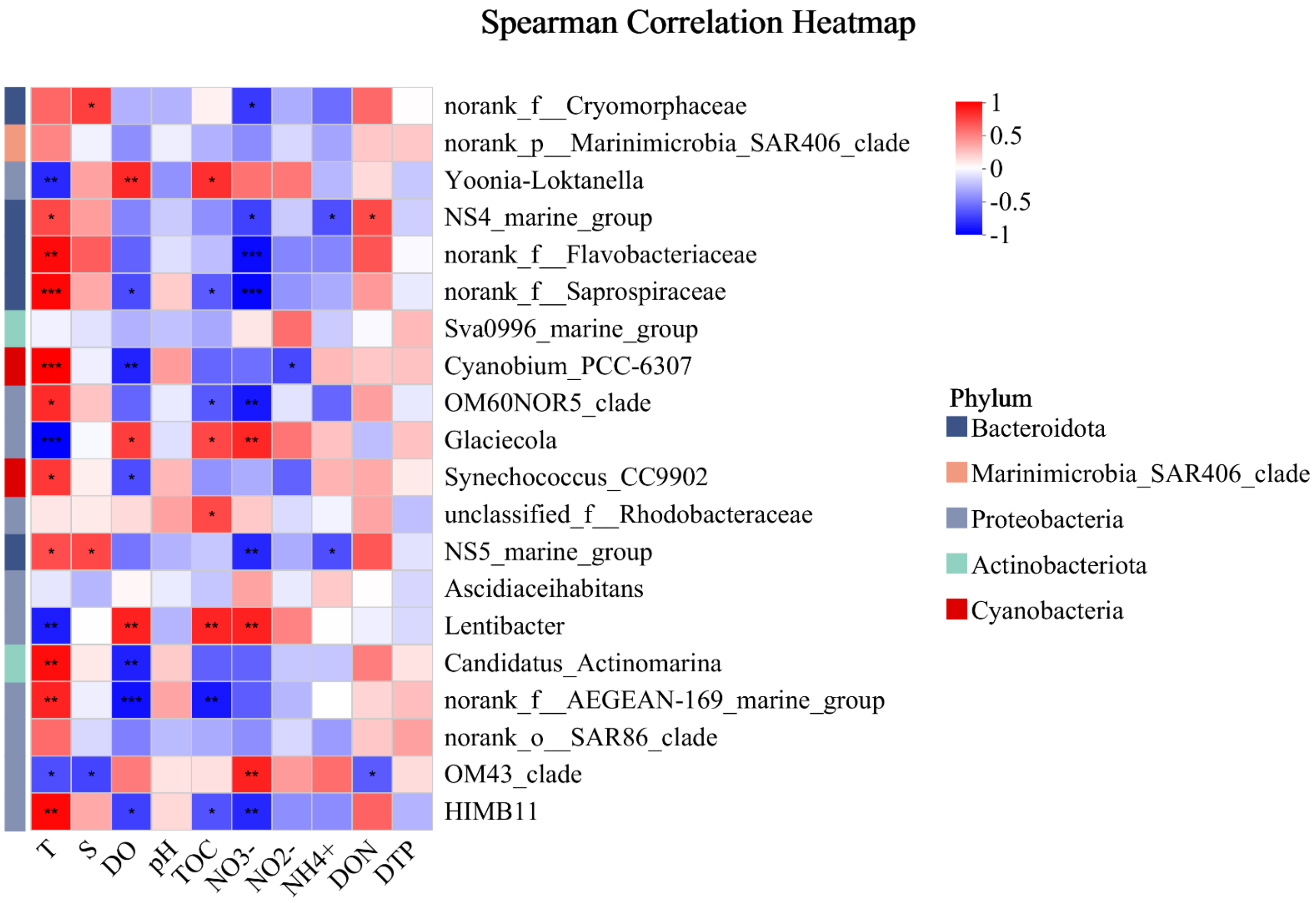

Figure S2. Heatmap of correlation analysis between species and environmental factors at the Genus level.
